# Usefulness of the neutrophil-to-lymphocyte ratio in predicting the severity of COVID-19 patients: a retrospective cohort study

**DOI:** 10.1590/1516-3180.2021.0298.R1.27052021

**Published:** 2021-07-30

**Authors:** Erdal Yılmaz, Rohat Ak, Fatih Doğanay

**Affiliations:** I MD. Specialist in Emergency Medicine, Department of Emergency Medicine, Kartal Dr. Lütfi Kırdar Şehir Hastanesi, Istanbul, Turkey.; II MD. Specialist in Emergency Medicine, Department of Emergency Medicine, Kartal Dr. Lütfi Kırdar Şehir Hastanesi, Istanbul, Turkey.; III MD. Specialist in Emergency Medicine, Department of Emergency Medicine, Edremit Devlet Hastanesi, Balıkesir, Turkey.

**Keywords:** COVID-19, Neutrophils, Lymphocytes, Intensive care units, Mortality, SARS-CoV-2, Survival, NLR, Neutrophil-to-lymphocyte ratio, Emergency

## Abstract

**BACKGROUND:**

Quick and accurate identification of critically ill patients ensures appropriate and correct use of medical resources. In situations that threaten public health, like pandemics, rapid and effective methods are needed for early disease detection among critically ill patients.

**OBJECTIVE:**

To determine the relationship between the neutrophil-to-lymphocyte ratio (NLR) of coronavirus disease-19 (COVID-19) patients upon admission to the emergency department (ED) and these patients’ prognosis.

**DESIGN AND SETTING:**

Retrospective cohort study among COVID-19 patients in the ED of a tertiary-level hospital.

**METHODS:**

Data on patients’ age, gender, vital signs, chronic diseases, laboratory tests and clinical outcomes were collected from electronic medical records. Receiver operating characteristic (ROC) curve analysis was performed. The area under the curve (AUC) was used to assess the accuracy of NLR for predicting in-hospital mortality risk and intensive care unit (ICU) requirement. The Youden J index (YJI) was used to determine optimal threshold values.

**RESULTS:**

1,175 patients were included. Their median age was 63 years (IQR, 48-75). With an NLR cutoff value of 5.14, the sensitivity, specificity, PPV, AUC and YJI for ICU requirement were calculated as 77.87%, 74.08%, 92.4%, 0.811 and 0.5194, respectively. With the same cutoff value, the sensitivity, specificity, AUC and YJI for in-hospital mortality were 77.27%, 75.82%, 0.815 and 0.5309, respectively. In addition, advanced age, leukocytosis, anemia and lymphopenia were found to be associated with poor prognosis.

**CONCLUSION:**

The NLR, which is a widely available simple parameter, can provide rapid insights regarding early recognition of critical illness and prognosis among COVID-19 patients.

## INTRODUCTION

In December 2019, a novel coronavirus named severe acute respiratory syndrome coronavirus 2 (SARS-CoV-2), was identified in Wuhan, China, and it spread rapidly all over the world.^1^ The World Health Organization (WHO) gave the name coronavirus disease-19 (COVID-19) to the resultant disease and declared it to be an epidemic.^2^ The first polymerase chain reaction (PCR)-positive COVID-19 case in Turkey was detected on March 11, 2020.^3^

In some studies in the literature, it was reported that 14% of COVID-19 patients developed severe pneumonia, one in 20 patients had an intensive care unit (ICU) requirement and approximately 66% of critically ill patients died.^4^^,^^5^^,^^6^ High mortality rates among critically ill patients and rapidly spreading disease raise concerns about ICU requirements, which may place pressure on healthcare system resources.^7^ In order to make optimal treatment decisions, prognostic predictors of mortality among COVID-19 patients need to be identified in order to help assess the severity of the condition.

Compared with the normal range, lower white blood cell (WBC) and lymphocyte counts but higher neutrophil counts have been found in COVID-19 patients.^8^^,^^9^ In some recent studies, it has been reported that the neutrophil-to-lymphocyte ratio (NLR) is an independent prognostic factor for predicting outcomes of critical illness and in-hospital mortality among COVID-19 patients.^10^^,^^11^

## OBJECTIVE

Our aim in this study was to determine the relationship between the prognosis and the NLR among COVID-19 patients.

## METHODS

This retrospective cohort study was carried out in the emergency department (ED) of Kartal Dr. Lütfi Kırdar City Hospital between October 1, 2020, and March 1, 2021. The hospital’s institutional review board approved the analysis and issued a waiver of consent (Ethics Committee Ruling number: 2021/514/198/30; date: March 29, 2021).

All COVID-19 patients over the age of 18 who were hospitalized between October 1, 2020, and March 1, 2021, were included in this study. The diagnosis of COVID-19 was determined based on the WHO guidelines. This study includes only patients who had positive results from a real-time reverse transcriptase-polymerase chain reaction (RT-PCR) test on nasal and pharyngeal swab samples.^12^ A data form was used to collect patients’ age, gender, vital signs, chronic diseases, laboratory tests and clinical outcome data from the electronic medical records.

### Measurements

Chronic diseases presented by these patients, such as chronic obstructive pulmonary disease (COPD), congestive heart failure (CHF), coronary artery disease (CAD), atrial fibrillation (AF), chronic renal failure (CRF), chronic neurological disease (CND), diabetes mellitus (DM) and hypertension (HT) were recorded by scanning digital file records stored in the hospital information management system (HIMS).

The following vital signs were assessed: systolic blood pressure (SBP), heart rate (HR) and blood oxygen saturation (SpO_2_); along with the following laboratory parameters: white blood cells (WBC), neutrophils (Neu), lymphocytes (Lym), monocytes (Mon), hemoglobin (Hgb) and platelets (Plt). These were measured in the ED.

The NLR was calculated using the following simple formula: Absolute number of neutrophils/Absolute number of lymphocytes.

### Outcomes

ED outcomes were determined as inpatient unit (IU) or ICU; and hospitalization outcomes as survivor or non-survivor.

### Data analysis

Statistical analyses were performed using the Statistical Package for the Social Sciences (SPSS) software (IBM Corp., released 2020; IBM SPSS Statistics for Windows, version 26.0; Armonk, NY, United States) and the MedCalc statistical software version 19.0.6 (MedCalc Software bvba, Ostend, Belgium; https://www.medcalc.org; 2019).

Continuous data did not meet the assumption of normality. The Mann-Whitney U test was used for analyses on continuous data and the chi-square test was used for analyses on categorical data. Continuous data were reported as medians and interquartile ranges (IQR; 25^th^ to 75^th^ percentile), while categorical data were reported as frequencies and percentages (Tables 1 and 2). A P-value of less than 0.05 was considered statistically significant.

**Table 1.** Gender and comorbidity data of the study population

		ICU requirement groups					Mortality groups				
	n = 1175	IU (n = 922)		ICU (n = 253)		Sig.	Survivor (n = 889)		Non-survivor (n = 286)		Sig.
Variables	Category	n	%	n	%	P	n	%	n	%	P
Sex	Male	483	79.6	124	20.4	0.341	469	77.3	138	22.7	0.185
	Female	439	77.3	129	22.7		420	73.9	148	26.1	
COPD	Absent	881	79.7	224	20.3	< 0.001	852	77.1	253	22.9	< 0.001
	Present	41	58.6	29	41.4		37	52.9	33	47.1	
DM	Absent	671	78.1	188	21.9	0.562	654	76.1	205	23.9	0.588
	Present	251	79.7	64	20.3		235	74.6	80	25.4	
HT	Absent	596	80	149	20	0.093	581	78.0	164	22.0	0.014
	Present	326	75.8	104	24.2		308	71.6	122	28.4	
CHF	Absent	883	81.2	204	18.8	< 0.001	856	78.7	231	21.3	< 0.001
	Present	39	44.3	49	55.7		33	37.5	55	62.5	
CAD	Absent	853	80.7	204	19.3	< 0.001	821	77.7	236	22.3	< 0.001
	Present	69	58.5	49	41.5		68	57.6	50	42.4	
AF	Absent	908	79.5	234	20.5	< 0.001	872	76.4	270	23.6	0.001
	Present	14	42.4	19	57.6		17	51.5	16	48.5	
CRF	Absent	865	79.5	223	20.5	0.002	848	77.9	240	22.1	< 0.001
	Present	57	65.5	30	34.5		41	47.1	46	52.9	
CND	Absent	888	79.3	232	20.7	0.002	865	77.2	255	22.8	< 0.001
	Present	34	61.8	21	38.2		24	43.6	31	56.4	

IU = inpatient unit; ICU = intensive care unit; COPD = chronic obstructive pulmonary disease; DM = diabetes mellitus; HT = hypertension; CHF = congestive heart failure; CAD = coronary artery disease; AF = atrial fibrillation; CRF = chronic renal failure; CND = chronic neurological disease; Sig. = significance.

**Table 2.** Descriptive statistics for age, vital parameters and laboratory measurements of the groups

		ICU requirement groups Median (IQR)			Mortality groups Median (IQR)		
Variables	All Patients (n = 1175)	IU (n = 922)	ICU (n = 253)	P	Survivor (n = 889)	Non-survivor (n = 286)	P
Age	63 (48-75)	59 (46-72)	74 (64-81)	< 0.001	58 (46-70)	75 (65-82)	< 0.001
Vital parameters							
SBP (mmHg)	120 (110-130)	120 (110-130)	120 (110-135)	0.278	120 (110-130)	120 (110-135)	0.256
HR (bpm)	85 (76-98)	83 (75-94)	98 (85-115)	< 0.001	83 (75-94)	96 (83-112)	< 0.001
SpO_2_ (%)	96 (93-98)	96 (94-98)	90 (84-96)	< 0.001	96 (94-98)	92 (85-96)	< 0.001
Laboratory measurements							
WBC (10^3^/ul)	6.3 (4.8-8.8)	5.9 (4.5-7.6)	9.6 (6.8-14)	< 0.001	5.8 (4.4-7.5)	9.4 (6.8-13.9)	< 0.001
Neu (10^3^/ul)	4.3 (3.0-6.8)	3.8 (2.7-5.4)	8.2 (5.4-12.1)	< 0.001	3.8 (2.7-5.3)	7.95 (5.20-12.03)	< 0.001
Lym (10^3^/ul)	1.1 (0.8-1.6)	1.2 (0.9-1.6)	0.8 (0.5-1.3)	< 0.001	1.2 (0.9-1.7)	0.8 (0.6-1.3)	< 0.001
Mon (10^3^/ul)	0.43 (0.3-0.6)	0.5 (0.3-0.6)	0.4 (0.2-0.7)	0.509	0.43 (0.3-0.6)	0.45 (0.30-0.70)	0.788
Hgb (g/dl)	12.7 (11.3-13.9)	13 (11.8-14)	11.6 (9.9-13.1)	< 0.001	13 (11.9-14)	11.5 (9.5-13)	< 0.001
Plt (10^3^/ul)	203 (158-255)	199 (157-199)	224 (163-288)	0.001	199 (156-248)	215 (161-277)	0.005
NLR	3.8 (2.2-7.8)	3.1 (1.9-5.5)	9.7 (5.7-16.9)	< 0.001	3.07 (1.93-5.03)	9.55 (5.58-15.63)	< 0.001

IU = inpatient unit; ICU = intensive care unit; IQR = interquartile range (25^th^ to 75^th^ percentile); SBP = systolic blood pressure; HR = heart rate; SpO_2_ = blood oxygen saturation; WBC = white blood cells; Lym = lymphocytes; Neu = neutrophils; Mon = monocytes; Hgb; hemoglobin; Plt = platelets, NLR = neutrophil-to-lymphocyte ratio.

Receiver operating characteristic (ROC) analysis was performed, in which the area under the curve (AUC) was calculated to evaluate the predictive accuracy of the NLR values by means of the DeLong method.^13^ The Youden J index (YJI) was used to calculate the highest threshold values for sensitivity, specificity, positive predictive value (PPV) and negative predictive value (NPV).^14^

## RESULTS

This study was conducted using data from 1,175 patients, among whom 439 were women and 483 were men. There were 889 patients in the survivor group and 286 patients in the non-survivor group; and there were 922 patients in the IU group and 253 patients in the ICU group (Table 1).

The median age of the population included in the study was 63 years (IQR: 48-75), with a minimum age of 19 and a maximum age of 98. The median ages in years (with IQR) of the groups were as follows: 58 (46-70) survivors and 75 (65-82) non-survivors; 59 (46-72) IU and 74 (64-81) ICU (Table 2).

Analysis on the effects of chronic diseases on the prognosis for COVID-19 showed that there were significant differences between the survivor and non-survivor groups and between the IU and ICU groups regarding COPD, CHF, CAD, AF, CRF and CND. While there was a significant difference between the survivor and non-survivor groups regarding hypertension (HT), there was no significant difference between the IU and ICU groups (Table 1). While there were statistically significant differences in both pairs of groups regarding HR and SpO_2_, which are among the vital signs, there was no statistically significant difference regarding SBP (Table 2). While significant differences (P < 0.001) were detected in both pairs of groups regarding WBC, Neu, Lym, Hgb, Plt and NLR among the laboratory parameters, there was no significant difference in either pair of groups regarding monocytes (Table 2). The data on the study population are presented in Tables 1 and 2.

**Figure 1.**
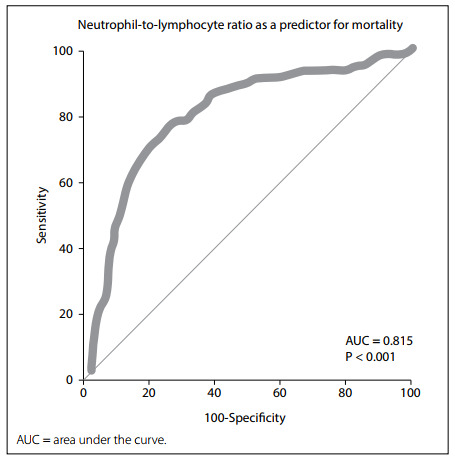
Neutrophil-to-lymphocyte ratio as a predictor for mortality.

**Figure 2.**
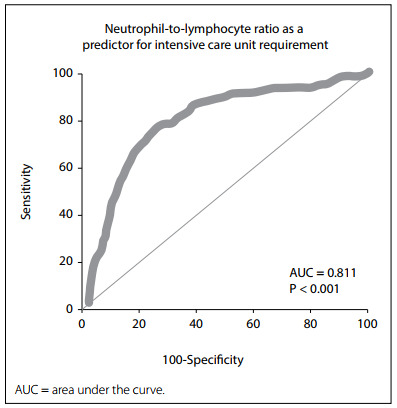
Neutrophil-to-lymphocyte ratio as a predictor for intensive care unit requirement.

Predictive values for NLR, in relation to in-hospital mortality and ICU requirement, were analyzed by means of ROC analysis. With an NLR cutoff value of 5.14, the sensitivity, specificity, PPV, NPV, AUC and YJI values for in-hospital mortality were calculated as 77.27%, 75.82%, 50.7%, 91.2%, 0.815 and 0.5309, respectively (P < 0.001) (Figure 1 and Table 3). With the same NLR cutoff value, the sensitivity, specificity, PPV, NPV, AUC and YJI values for ICU requirement were calculated as 77.87%, 74.08%, 45.2%, 92.4%, 0.811 and 0.5194, respectively (P < 0.001) (Figure 2 and Table 3).

**Table 3.** Predictive accuracy of neutrophil-to-lymphocyte ratio in terms of severity among COVID-19 patients

Sensitivity	Specificity	PPV	NPV	AUC (95% CI)	YJI	Criterion for YJI	P
As a predictor for intensive care unit requirement							
77.87	74.08	45.2	92.4	0.811 (0.788 - 0.833)	0.5194	> 5.143	< 0.001
As a predictor for mortality							
77.27	75.82	50.7	91.2	0.815 (0.792 - 0.837)	0.5309	> 5.143	< 0.001

NLR = neutrophil-to-lymphocyte ratio; AUC = area under the curve; PPV = positive predictive value; NPV = negative predictive value; YJI = Youden J index; CI = confidence interval.

## DISCUSSION

COVID-19 has a significant impact on the hematopoietic system. Dysregulation of the hematological and immunological systems plays a key role in the pathological process of this infection.^15^

In this study, we concluded that the NLR values of COVID-19 patients at the time of admission to the ED can be used as a predictor for ICU requirement and mortality risk. In addition, advanced age, leukocytosis, anemia and lymphopenia were found to be associated with poor prognosis. Over recent years, the diagnostic and prognostic accuracy of various ratios such as neutrophil-to-lymphocyte, thrombocyte-to-lymphocyte and monocyte-to-lymphocyte have been studied in relation to many inflammatory conditions. Similar studies are ongoing in the COVID-19 pandemic.

In a study based on retrospective analysis of clinical data from 443 patients with COVID-19, Shang et al. reported that NLR, C-reactive protein (CRP) and platelet values can help determine the severity of the disease. They also reported that although all these parameters have prognostic significance, NLR had the best predictive performance.^16^

In a recent study conducted in Turkey, the relationship between ICU requirement for COVID-19 patients and their hemogram parameters at the time of initial admission was investigated. It was highlighted that high NLR and monocyte-to-lymphocyte and low platelet-to-lymphocyte ratios can be predictors for ICU requirement.^17^

In a prospective study conducted in Pakistan, it was reported that use of the NLR successfully enabled early recognition of severe conditions among patients with COVID-19 pneumonia.

In a study assessing the prognostic accuracy of the NLR, the AUC was calculated as 0.831 and the YJI was 0.589. In addition, it was reported that, for the optimum NLR threshold value of 4.795, the sensitivity was 83.9% and the specificity was 75%.^18^ In our study, the best threshold value was found to be 5.14. In terms of ICU requirement, we calculated the sensitivity as 77.87%, specificity 74.08%, AUC 0.811 and YJI 0.519 for this NLR cutoff value of 5.14. Evaluation of the same cutoff value in terms of mortality prediction gave rise to sensitivity calculated as 72.27%, specificity 50.7%, AUC 0.815 and YJI 0.530.

The immune system is the system that is most affected by COVID-19 infection, after the respiratory system.^19^ Therefore, it is not surprising that the NLR has high predictive accuracy. In assessing the pathogenesis of the disease, it is seen that necrosis, bleeding and atrophy occur in the spleen, and also that there are significant decreases in lymphocyte and neutrophil counts. In addition, in cases in which the inflammatory response increases, lymphocytes in lymph nodes are depleted and the numbers of CD4+ and CD8+ cells decrease.^20^

While neutrophils are vital in the innate immune response, lymphocytes also play an important role in the adaptive inflammatory response. Therefore, increased NLR reflects the imbalance of the inflammatory response and can be considered to be a possible indicator of disease severity in infectious diseases such as sepsis and bacteremia.^21^ In a meta-analysis examining 15 studies, it was reported that the neutrophil count and NLR were higher, but that the lymphocyte count was lower in severe COVID-19 cases, compared with non-severe cases.^22^ Additionally, recent studies have reported that NLR can be a reliable predictor, not only for inflammatory diseases and infections, but also for other acute medical conditions, including cerebral hemorrhage, acute coronary syndrome and ischemic stroke.^23^^,^^24^^,^^25^

In general, elderly patients have been shown to be the “most vulnerable” group with regard to COVID-19 mortality.^26^^,^^27^ In one study, COVID-19 patients aged 60 years and over were shown to have greater severity of clinical outcomes and higher mortality rates, compared with those who were under 60.^28^ Similarly, in our study, we concluded that greater age was associated with increased risk of ICU requirement and mortality.

This study had certain limitations, such as having a relatively small sample size and being a single-center study. For more accurate and precise results, wider generalizability of the findings and validation of our results, multicenter clinical studies with larger sample sizes are required.

## CONCLUSION

The NLR, which is a widely available simple parameter, can provide rapid insights regarding early recognition of critical illness and prognosis among COVID-19 patients.
